# Differential regulation of blood flow‐induced neovascularization and mural cell recruitment by vascular endothelial growth factor and angiopoietin signalling

**DOI:** 10.1113/JP273430

**Published:** 2017-02-02

**Authors:** Oliver A. Stone, James G. Carter, P. Charles Lin, Ewa Paleolog, Maria J. C. Machado, David O. Bates

**Affiliations:** ^1^ Microvascular Research Laboratories, Bristol Heart Institute, School of Physiology and PharmacologyUniversity of BristolBristolUK; ^2^ Center for Cancer ResearchNational Institute of CancerFrederickMD2170USA; ^3^ Kennedy Institute of RheumatologyUniversity of Oxford65 Aspenlea RoadHammersmithLondonUK; ^4^ Cancer Biology, Division of Oncology, School of Clinical Sciences, University of NottinghamQueen's Medical CentreNottinghamUK

**Keywords:** angiogenesis, angiopoitin, arteriolargenesis, neovascularisation, VEGF

## Abstract

**Key points:**

- Combining nitric oxide (NO)‐mediated increased blood flow with angiopoietin‐1–Tie2 receptor signalling induces arteriolargenesis – the formation of arterioles from capillaries – in a model of physiological angiogenesis.
- This NO–Tie‐mediated arteriolargenesis requires endogenous vascular endothelial growth factor (VEGF) signalling.
- Inhibition of VEGF signalling increases pericyte coverage in microvessels.
- Together these findings indicate that generation of functional neovasculature requires close titration of NO–Tie2 signalling and localized VEGF induction, suggesting that the use of exogenous VEGF expression as a therapeutic for neovascularization may not be successful.

**Abstract:**

Signalling through vascular endothelial growth factor (VEGF) receptors and the tyrosine kinase with IgG and EGF domains‐2 (Tie2) receptor by angiopoietins is required in combination with blood flow for the formation of a functional vascular network. We tested the hypothesis that VEGF and angiopoietin‐1 (Ang1) contribute differentially to neovascularization induced by nitric oxide (NO)‐mediated vasodilatation, by comparing the phenotype of new microvessels in the mesentery during induction of vascular remodelling by over‐expression of endothelial nitric oxide synthase in the fat pad of the adult rat mesentery during inhibition of angiopoietin signalling with soluble Tie2 (sTie2) and VEGF signalling with soluble Fms‐like tyrosine kinase receptor‐1 (sFlt1). We found that NO‐mediated angiogenesis was blocked by inhibition of VEGF with sFlt1 (from 881 ± 98% increase in functional vessel area to 279 ± 72%) and by inhibition of angiopoietin with sTie2 (to 337 ± 67%). Exogenous angiopoietin‐1 was required to induce arteriolargenesis (8.6 ± 1.3% of vessels with recruitment of vascular smooth muscle cells; VSMCs) in the presence of enhanced flow. sTie2 and sFlt1 both inhibited VSMC recruitment (both 0%), and VEGF inhibition increased pericyte recruitment to newly formed vessels (from 27 ± 2 to 54 ± 3% pericyte ensheathment). We demonstrate that a fine balance of VEGF and angiopoietin signalling is required for the formation of a functional vascular network. Endogenous VEGF signalling prevents excess neovessel pericyte coverage, and is required for VSMC recruitment during increased nitric oxide‐mediated vasodilatation and angiopoietin signalling (NO–Tie‐mediated arteriogenesis). Therapeutic vascular remodelling paradigms may therefore require treatments that modulate blood flow to utilize endogenous VEGF, in combination with exogenous Ang1, for effective neovascularization.

## Key points

- Combining nitric oxide (NO)‐mediated increased blood flow with angiopoietin‐1–Tie2 receptor signalling induces arteriolargenesis – the formation of arterioles from capillaries – in a model of physiological angiogenesis.
- This NO–Tie‐mediated arteriolargenesis requires endogenous vascular endothelial growth factor (VEGF) signalling.
- Inhibition of VEGF signalling increases pericyte coverage in microvessels.
- Together these findings indicate that generation of functional neovasculature requires close titration of NO–Tie2 signalling and localized VEGF induction, suggesting that the use of exogenous VEGF expression as a therapeutic for neovascularization may not be successful.

**Abbreviations**
Ad: adenovirus
Ang1: angiopoietin‐1
EC: endothelial cell
eGFP: enhanced green fluorescent protein
ELISA: enzyme linked immunosorbent assay
eNOS: endothelial nitric oxide synthase
Flt1: Fms‐like tyrosine kinase receptor‐1
FVA: functional vessel area
GFP: green fluorescent protein
NG2: neuro/glial antigen 2
NO: nitric oxide
PDGF: platelet derived growth factor
PlGF: placental growth factor
sFlt1: soluble Flt1
SMA: smooth muscle actin
sTie2: soluble Tie2
Tie2: tyrosine kinase with IgG and EGF domains‐2
VEGF: vascular endothelial growth factor
VEGFR: VEGF receptor
VSMC: vascular smooth muscle cell

## Introduction

Chronic occlusive vascular disorders represent a significant hurdle in global healthcare. Although advances in the field of interventional medicine have significantly improved clinical outcome, a considerable proportion of patients cannot be managed adequately by ‘traditional’ therapies. For these patients, therapeutic induction of blood vessel growth remains an attractive treatment option (Gupta *et al*. 2009). However, translation from pre‐clinical studies to clinical practice has been limited (Laitinen *et al*. 2000; Comerota *et al*. 2002; Grines *et al*. 2002), possibly reflecting the inability of any single factor to induce the growth of a complete, functional vascular network. Regulated perfusion requires the formation of capillary, arterial and venous networks. Accurately defining the molecular and physical signals that regulate neovascularization from endothelial sprouting through to arteriogenesis could facilitate the future development of therapeutics.

The vascular endothelial growth factor (VEGF)–VEGF receptor (VEGFR) and angiopoietin (Ang)–tyrosine kinase with IgG and EGF domains‐2 (Tie2) signalling axes differentially regulate neovascularization and vessel maturation. VEGF‐A orchestrates the initial formation of blood vessels and is a potent endothelial cell mitogen (Carmeliet *et al*. 1996; Ferrara *et al*. 1996), while angiopoietin signalling appears to regulate endothelial cell quiescence and mural cell recruitment (Maisonpierre *et al*. 1997; Thurston *et al*. 1999). The detailed contribution of VEGF‐A signalling to neovessel maturation is ambiguous. Excessive VEGF‐A expression is a hallmark of many solid tumours and stimulates the formation of tortuous, leaky microvessels, which lack mural cell investment (Willett *et al*. 2004; Winkler *et al*. 2004). For instance, in mouse models of glioblastoma, inhibition of VEGFR2 signalling can stimulate Ang1‐dependent pericyte recruitment and enhance perfusion (Winkler *et al*. 2004). In contrast, intravitreal injection of VEGF‐A_165_ in a murine neonatal model was shown to completely prevent vessel regression by stimulating pericyte recruitment (Benjamin *et al*. 1998). When given alone, VEGF‐A_165_ overexpression has been shown to enhance arterial remodelling, or arteriogenesis, in rabbit models of hindlimb ischaemia (Rissanen *et al*. 2005), and endogenous VEGF‐A is required for collateral artery development during ischaemia (Clayton *et al*. 2008) indicating that VEGF is required, but its role, source and/or localization may all be critical. We previously found that, in the presence of increased blood flow, a combination of exogenous vascular growth factors (VEGF‐A_165_ and Ang1) could induce arterial remodelling in a model – the rat mesenteric angiogenesis assay – that can delineate the molecular and physical control of both angiogenesis and capillary arterialization (or arteriolargenesis) in adult animals (Benest *et al*. 2006, 2008). In this model, adenoviral overexpression of a gene of interest induces localized blood vessel growth into mesenteric connective tissue panels, which, under normal conditions, are sparsely vascularized and lack vascular smooth muscle cells (VSMCs; αSMA^+^ cells). The advantages of using the mesenteric assays are that it is an easily visualized, two‐dimensional network, which allows the intravital recording of blood flowing through arteriolar, true and venular capillaries, molecular analysis of vessel architecture and networks, and visualization of the same tissue on two different days in an adult, essentially quiescent, vasculature (Benest & Bates, 2016). It also allows the modification of both the physiology and the growth and maturation of vessels by using the addition of secreted growth factors or systemic agents. Using this assay we demonstrated that increased blood flow following enhanced nitric oxide production by overexpression of endothelial nitric oxide synthase with adenoviruses (Ad.*eNOS*) or by administration of a vasodilator (prazosin) stimulates angiogenesis and upregulated endogenous VEGF‐A and Ang1 (Benest *et al*. 2008). Ad.*eNOS* induced the same changes in the mesenteric microcirculation (vasodilatation and smooth muscle cell recruitment in the presence of VEGF and Ang1) as continuous systemic administration of prazosin, but with an effect that acts only locally to the mesenteric panel, not systemically altering haemodynamics. Following treatment with Ad.*eNOS*, and adenoviruses expressing VEGF‐A_165_ (Ad.*VEGF*) and angiopoietin‐1 (Ad.*Ang1*) we observed recruitment of VSMCs and the formation of arterioles (Benest *et al*. 2008), demonstrating that exogenous growth factor expression in combination with enhanced blood flow can stimulate arteriolargenesis in a non‐ischaemic setting. However, the relative contributions of exogenous and endogenous VEGF‐A_165_ and Ang1 were unclear.

We therefore tested the hypothesis that endogenous VEGFs and Ang signalling contribute differentially to the process of vascular maturation induced by increased blood flow, using Ad.*eNOS*‐induced neovascularization as a model of blood flow‐induced neovascularization. Using an inhibitor of VEGF (Ad.*sFlt1*) and Ang (Ad.*sTie2*) we were able to demonstrate contrasting roles in vessel growth and maturation for these two signalling pathways. Whilst *Ad.sTie2* is specific for Ang1 signalling, Ad.*sFlt1* is predicted to block VEGF‐A_165_ signalling, as well as all of the other VEGF‐A isoforms, along with placental growth factor (PlGF) and VEGF‐B. Thus, we further report that the ambiguous nature of VEGF–VEGFR signalling in the control of neovessel maturation can be partly explained by the contribution of endogenous VEGFs.

## Methods

### Ethical approval

Animal experiments were carried out under UK Home Office licence under the Animal (Scientific procedures) Act, after review by the local ethical review board and experiments were carried out according to the guidelines laid down by the institution's animal welfare committee, and conform to the principles and regulations, as described in the Editorial by (Grundy, 2015).

### Adenoviruses

Ad.*VEGF* was previously described and shown to give rise to the over‐expression of the human VEGF‐A_165_ isoform in the mesentery (Wang *et al*. 2004). Ad.*Ang1* and Ad.*eGFP* (enhanced green fluorescent protein) were a gift from Regeneron Inc., Tarrytown, NY, USA (Benest *et al*. 2006) and Ad.*eNOS* (endothelial nitric oxide synthase) from Prof. Keith Channon, University of Oxford (Benest *et al*. 2008); Ad.*sFlt1* (soluble Fms‐like tyrosine kinase receptor‐1/soluble VEGFR1) was generated by Dr Ewa Paleolog, Imperial College London (Afuwape *et al*. 2003) and Ad.*sTie2* (soluble Tie2) (Lin *et al*. 1998) by Prof. Charles Lin, Vanderbilt University, Nashville, TN, USA. Plaque purification was used to ensure biological activity. To validate Ad.*sFlt1* and Ad.*sTie2* in the mesenteric assay, these adenoviruses and Ad.*eGFP* control were injected in perfused mesenteric panels of rats (see protocol for assay below). The tissue was excised 24, 48 or 72 h later, and snap‐frozen in liquid nitrogen. Tissue was crushed under liquid nitrogen into a fine powder, and the liquid nitrogen allowed to evaporate; 500 μl of RIPA buffer (supplemented with 1 mm phenylmethanesulphonylfluoride, 1 mm sodium orthovanadate, 20 μg ml^–1^ aprotinin, 10 μg ml^–1^ leupeptin, 10 μg ml^–1^ pepstatin) was then added and the lysate incubated on ice for 20–25 min with occasional agitation. Lysates were centrifuged for 10 min at 4°C and 13500 *g*; then the supernatant was removed and stored at –20°C. Protein concentration was determined by the Bradford assay. Standard SDS‐PAGE and Western blotting was used with 30 μg tissue loaded per well. Western blots were probed with rabbit monoclonal antibody to human sFlt1 (1:10,000; ab32152; Abcam, Cambridge, UK) or a mouse monoclonal antibody to human Tie2 (2 μg ml^–1^; 334201; Biolegend, London, UK). Enzyme linked immunosorbent assay (ELISA) was used for determination of VEGF‐A and Ang1. Quantikine® ELISA kits were used for measurement of human Ang1 (DANG10; R&D systems, Abingdon, UK). VEGF‐A was measured using a DuoSet VEGF‐A ELISA (DY293BE; R&D Systems). The ELISA was carried out according to the manufacturer's instructions and optical density of each well was determined with a microplate reader (Dynex Technologies, Worthing, UK) set at 450 nm with correction set at 570 nm.

### Mesenteric angiogenesis assay

**Figure 1:**
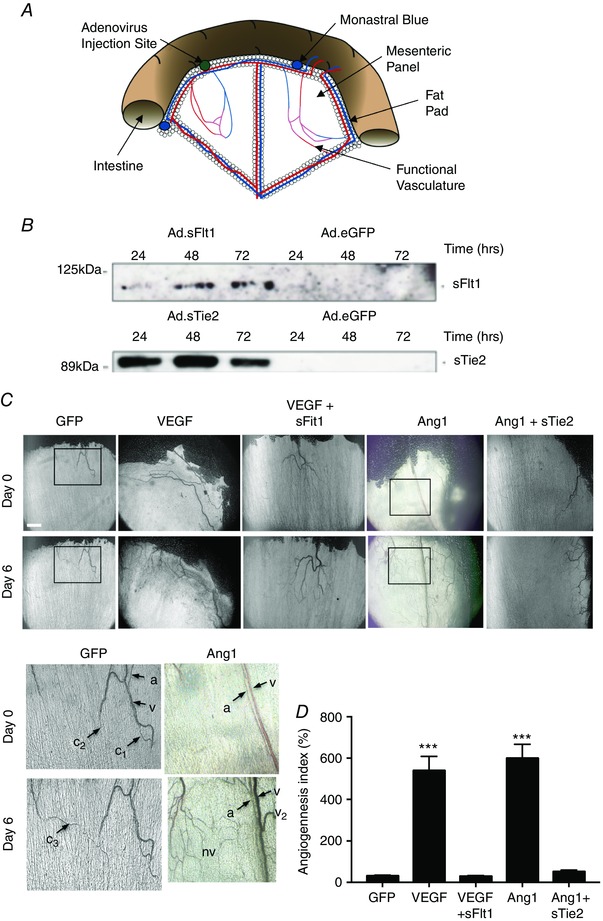
Expression of soluble receptors for VEGF and Tie2 inhibits VEGF‐ and Ang1‐mediated angiogenesis, respectively *A*, diagram of the mesenteric angiogenesis assay. The ileum was teased out of the abdominal cavity and vessels were imaged in the mesenteric connective tissue panel and then adenovirus injected into the fat pad. The site was marked with a small injection of Monastral Blue, and replaced in the animal. After 24, 48 or 72 h, the panel was found again and the tissue frozen for protein extraction; alternatively, 6 days later the panel was re‐imaged, fixed and stained for vascular markers. *B*, Western blots of sFlt1 and sTie2 expression after injection with the adenoviruses. *C*, images of mesenteries on day 0 and day 6. Scale bar: 1 mm. Higher power images of the GFP and Ang1 infected mesenteries from the boxes in the low power images are shown below. a, arteriole; c, capillary with numbers indicating how many were visible with flowing blood; nv, newly patent vessels; v, venule; v_2_, venule that was patent on day 6 but not on day 0. *D*, quantification of perfused vessel area. Changes in functional vessel area from day 0 to day 6 are expressed as the angiogenesis index (AI; %).

The rat mesenteric angiogenesis assay (Fig. 1) was used to characterize the neovessel phenotype as previously described (Wang *et al*. 2004; Benest *et al*. 2008; Stone *et al*. 2009). Male Wistar rats (300–350 g: typically 5–6 weeks old, Harlan, UK) were anaesthetized with 3% isoflurane vaporized in 100% oxygen and a laparotomy performed under sterile conditions, with depth of anaesthesia monitored by breathing rate, eyelid reflex and/or toe‐pinch reflex. While externalized, the mesentery and small intestine were constantly superfused with mammalian Ringer solution, which allowed for the mesenteric panels to be imaged intravitally using a ×4 objective on a Leica DMIL inverted microscope, and blood flow recorded by video microscopy onto S‐VHS tape. Adenoviruses expressing eGFP, eNOS, VEGF‐A_165_, Ang1, sFlt1 or sTie2 were injected into the mesenteric fat pad using a 30‐gauge needle and Hamilton syringe, which has been previously shown to result in infection of adipocytes (Wang *et al*. 2004). Adjacent panels were subsequently tattooed with Monastral Blue (0.6% w/v in mammalian Ringer solution), enabling recognition of the panel 6 days later. The animal was sutured and allowed to recover with analgesia provided by intra‐muscular injection of 0.3 mg kg^−1^ buprenorphine. Six days later the animals were anaesthetized using the same regimen as described previously (3% isoflurane vaporized in 100% oxygen) and the same mesenteric panel was located and imaged intravitally as before using a ×4 objective on a Leica DMIL, and blood flow through the microcirculation recorded by videomicroscopy. Animals were killed by cervical dislocation after the tissue was fixed *in situ* with 4% paraformaldehyde in PBS. The mesentery was then post‐fixed in 4% paraformaldehyde and immunofluorescence staining performed in the whole panel. The mesenteric fat pad was excised separately and protein extracted for Western blotting as previously described (Benest *et al*. 2008). The area of the mesenteric panel covered by vessels in which blood flow was occurring was determined from the intravital videomicroscopy by highlighting vessels in which blood flow was visible using Openlab software wand function (Improvision, Coventry UK). The fractional vessel area (area of flowing vessels as a proportion of the mesenteric connective tissue being imaged) was determined for days 6 and 0 and the ratio of the difference between these and day 0 was expressed as the angiogenesis index (%) (Wang *et al*. 2004).

### Immunofluorescence and image analysis

Mesenteric panels were washed in 0.5% phosphate buffered saline with 0.5% Triton X‐100 (PBX) (pH 6.8) and incubated with biotinylated isolectin‐B_4_ (10 μg ml^–1^; *Griffonia simplicifolia*; I‐21414; Invitrogen, Carlsbad, CA, USA) at 4°C overnight. The following day, tissue was washed and incubated with AF555‐streptavidin (2 μg ml^–1^; S‐21381; Invitrogen) for 2 h at room temperature, to stain for blood vessels. All other stains were achieved in parallel after blocking for 1 h in 1% BSA–0.5% PBX (pH 7.4) at room temperature. Tissue was incubated overnight at 4°C in block solution containing mouse monoclonal antibodies to either neuro/glial antigen 2 (NG2; 1:200; MAB5384; Millipore, Billerica, MA, USA) or Ki67 (1:200; NCL‐L‐Ki67‐MM1; Leica, Milton Keynes, UK), or rabbit polyclonal antibodies to α‐smooth muscle actin (1:125; ab5694; AbCam, UK) or NG2 (1:200; AB5320; Millipore, USA). The following day, panels were washed for 6 × 10 min in 0.5% PBX (pH 7.4) and secondary antibodies (goat anti‐mouse AF488: A11039, or goat anti‐rabbit AF350: A11046, used at 5 μg ml^–1^; Molecular Probes, UK) incubated in block solution for 2 h at room temperature. After secondary antibody incubation, panels were washed and incubated with Hoechst 33324 (1 μm; Molecular Probes, UK) for 10 min to stain mesenteric nuclei. Panels were mounted whole using Vectashield (Vector Laboratories, Peterborough, UK). Blood vessels were imaged by confocal microscopy and blinded analysis was carried out using Openlab. Counts of five random ×40 microscopic fields per mesenteric panel were averaged per animal (5–6 animals per group were used). Individual vessels were numbered, and measurements obtained for vessel diameter and length. The number of sprout points, branch points, and, in Ki67‐stained images, proliferating endothelial cell (EC) number, were counted and expressed as density per area of image. Vessels < 16 μm diameter were termed exchange vessels, while 16–35 μm diameter vessels were termed conduit vessels, as previous work has shown that vessels with diameters greater than 16 μm make very little contribution to solute exchange (Benest & Bates, 2009). Relative pericyte area was calculated as the mean percentage NG2 coverage of each individual vessel, while relative VSMC coverage was calculated as the percentage α‐smooth muscle actin (αSMA) coverage of all vessels measured.

### Statistical analysis

Results are expressed as means ± SEM. Comparisons between means of data were performed using one‐way ANOVA. *P* < 0.05 was considered statistically significant and the Holm–Sidak *post hoc* test was used to compare individual groups.

## Results

### Soluble receptor overexpression blocks Ad.*VEGF*‐ and Ad.*Ang1*‐induced neovascularization

To confirm the *in vivo* activity of the inhibitors of VEGF signalling (soluble Flt1, Ad.*sFlt1*) and angiopoietin 1 signalling (soluble Tie2, Ad.*sTie2)*, Ad.*VEGF*/Ad.*sFlt1* and Ad.*Ang1*/Ad.*sTie2* were administered in the mesenteric assay (Fig. 1 *A*). This infection dose of Ad.*VEGF* and Ad.*Ang1* results in 200–260 pg mg^−1^ VEGF and 13–18 pg mg^−1^ Ang1 in the mesenteric fat pad, as previously described (Benest *et al*. 2008). Both growth factors also independently induce vessel growth (Fig. 1 *C* and *D*). Ad.*sFlt1* and Ad.*sTie2*, both of which were expressed in adipocytes within 24 h after infection (Fig. 1 *B*), blocked the increase in blood vessels that could be visualized by intravital microscopy 6 days after infection (Fig. 1 *C*). This was determined by decreased functional vessel area (FVA) with respect to their controls (Fig. 1 *D*).

**Figure 2:**
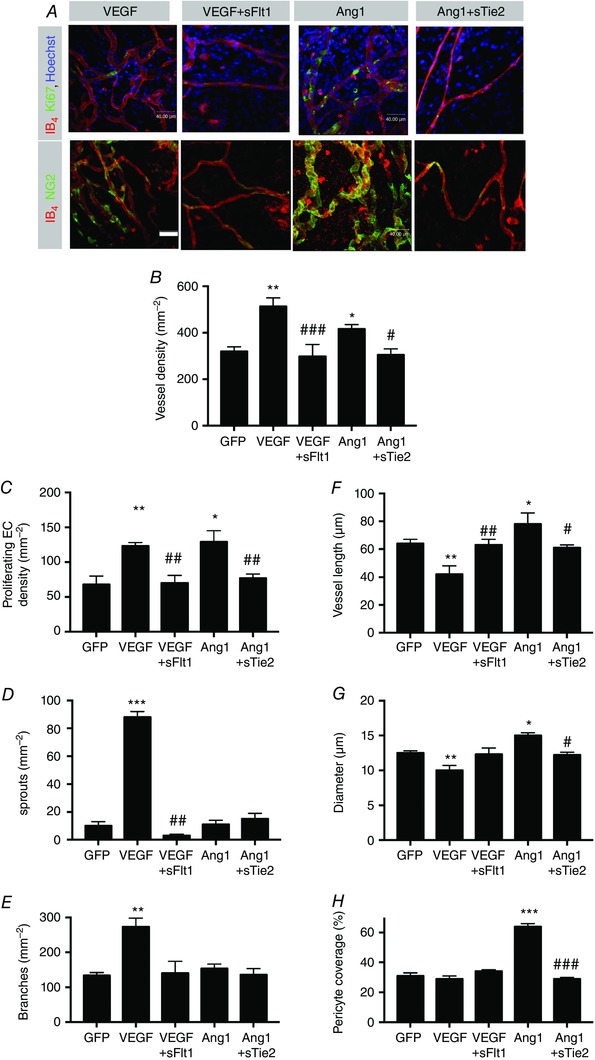
Soluble receptors for VEGF and Tie2 inhibit VEGF‐ and Ang1‐mediated markers of angiogenesis, respectively *A*, representative images of mesenteric panels stained for endothelial cells (IB4) and proliferating cells (Ki67). *B–H*, quantification of vessel density (*B*), proliferating endothelial cells (*C*), sprouts (*D*), branches (*E*), vessel length (*F*), vessel diameter (*G*) and pericyte coverage (*H*). ^*^ *P* < 0.05, ^**^ *P* < 0.01, ^***^ *P* < 0.001: significantly different from GFP; ^#^ *P* < 0.05, ^##^ *P* < 0.01, ^###^ *P* < 0.001: significantly different from agonist (VEGF or Ang1). Scale bar: 40 μm.

Staining of vessels for endothelial cells, proliferating endothelial cells and pericytes (Fig. 2 *A*) demonstrated that sFlt1 and sTie2 inhibited, respectively, the VEGF‐ and Ang1‐mediated increase in both vessel density (Fig. 2 *B*) and proliferating endothelial cell density (Fig. 2 *C*). sFlt1 inhibited VEGF‐induced sprouting (Fig. 2 *D*) and branching (Fig. 2 *E*), and sFlt1 and sTie2 reversed the effect of VEGF (a reduction relative to control) and Ang1 (an increase relative to control), respectively, on both vessel length (Fig. 2 *F*) and diameter (Fig. 2 *G*). sTie2 also reduced the increased pericyte coverage induced by Ang1 (Fig. 2 *H*). These results indicate that adenovirus over‐expression of sFlt1 and sTie2 was able to completely abrogate the responses of the mesentery induced by adenovirus‐mediated over‐expression of VEGF and Ang1, respectively. These results confirm that the soluble receptors are able to inhibit the effect of each of their cognate growth factors on neovascularization.

### Nitric oxide‐induced neovascularization requires VEGF–VEGFR and Ang–Tie signalling

**Figure 3:**
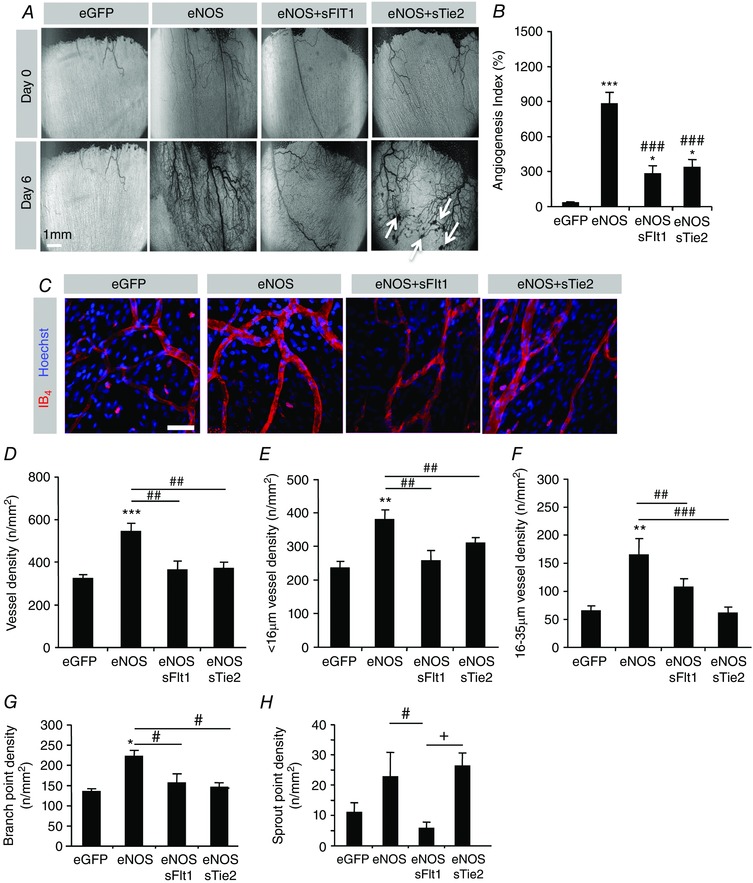
The blood flow‐dependent increase in angiogenesis is differentially inhibited by blockade of endogenous VEGF or endogenous Ang1 *A*, images of mesenteries after treatment with Ad.*eNOS* and VEGF or Ang1 inhibitors. Scale bar: 1 mm. *B*, the angiogenesis index indicates that Ad.*sFt1* and Ad.*sTie2* were both able to decrease the angiogenic response mediated by Ad.*eNOS*. *C*, confocal stack images from mesenteric panels stained with isolectin B4 and Hoechst 33324. *D–F*, analysis of vessel density (*D*), exchange vessel density (*E*), conduit vessel density (*F*), branch point density (*G*) and sprout point density (*H*). ^*^ *P* < 0.05, ^**^ *P* < 0.01, ^***^ *P* < 0.001 compared with GFP; ^#^ *P* < 0.05, ^##^ *P* < 0.01 compared with eNOS; ^++^ *P* < 0.01 compared with eNOS+sFlt1. Scale bar: 40 μm.

To determine the relative contribution of VEGF and Ang1 signalling in NO‐mediated angiogenesis, we first assessed vessel perfusion by measuring patent vessel area in individual mesenteric panels (Fig. 3 *A*). Exogenous addition of Ad.*sFlt1* or Ad.*sTie2* to Ad.*eNOS* in the mesenteric fat pad inhibited angiogenesis, as measured by the angiogenesis index (AI; Fig. 3 *B*). Interestingly, minor micro‐haemorrhages were observed following Ad.*eNOS*/Ad.*sTie2* treatment (Fig. 3 *A*), indicating that Ang–Tie signalling is required for maintenance of vessel integrity during NO‐induced remodelling.

### The architecture of NO‐induced neovascular networks depends upon VEGF–VEGFR and Ang–Tie signalling

We next evaluated the effect of blockade of VEGF–VEGFR and Ang–Tie signalling on vessel architecture. Vessel phenotype was analysed using confocal microscopy of mesenteric panels stained using isolectin B4 and counterstained with Hoechst 33324 (Fig. 3 *C*). Ad.*sFlt1* or Ad.*sTie2* inhibited NO‐mediated increases in vessel density (Fig. 3 *D*), of both exchange vessels (Fig. 3 *E*) and conduit vessels (Fig. 3 *F*), although sFlt1 was less effective at reducing the increase in larger vessels, although not significantly different from sTie2. Ad.*sFlt1* or Ad.*sTie2* also inhibited increases in branching (Fig. 3 *G*). However, sprouting was only inhibited by Ad.*sFlt1* and not by Ad.*sTie2* (Fig. 3 *G*). This suggests that VEGF–VEGFR and Ang–Tie signalling have different effects in NO‐mediated vessel patterning.

**Figure 4:**
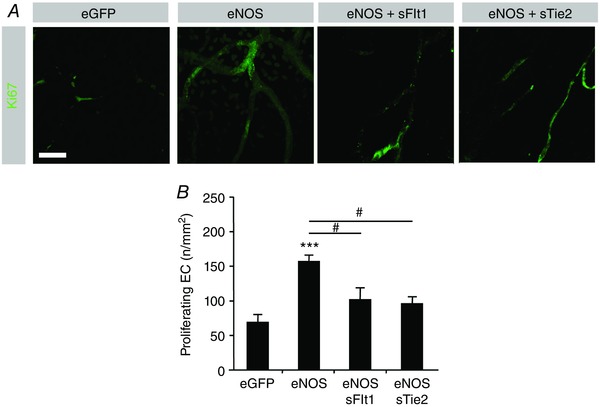
VEGF–VEGFR or Ang–Tie2 inhibition inhibits NO‐mediated endothelial proliferation *A*, confocal stack images from rat mesentery stained with antibodies for Ki67. *B*, proliferating endothelial cell density. ^***^ *P* < 0.001 compared with GFP; ^#^ *P* < 0.05 compared with eNOS. Scale bar: 40 μm.

**Figure 5:**
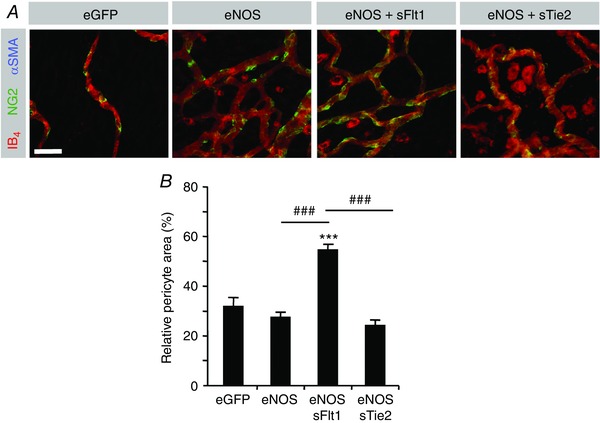
The VEGF–VEGFR and Ang–Tie signalling axes differentially regulate pericyte recruitment *A*, sample confocal stack images from rat mesentery 6 days post‐treatment stained with isolectin‐B_4_ (red) an antibody for NG2 (green) and for smooth muscle actin (blue). Pericytes were stained for NG2 but not SMA. No smooth muscle cells were seen in these mesenteries. *B*, relative pericyte area. Scale bar: 40 μm. ^***^ *P* < 0.001 *vs*. eGFP. ^###^ *P* < 0.001 *vs*. eNOS+sFlt1.

Both Ad.*sFlt1* and Ad.*sTie2* inhibited proliferation of endothelial cells at the same level (Fig. 4 *A* and *B*). The relative coverage of vessels by pericytes (Fig. 5 *A*) was not affected by NO‐mediated angiogenesis as the increase in vessel growth was matched by the increase in pericyte growth. No vascular smooth muscle cells were seen in the presence of eNOS and either sFlt1 or sTie2. However, treatment of mesenteric tissue with Ad.*sFlt1* significantly increased the pericyte coverage, indicating that endogenous VEGF inhibited pericyte growth (Fig. 5 *B*).

### Angiopoietin does not further enhance NO‐mediated angiogenesis

**Figure 6:**
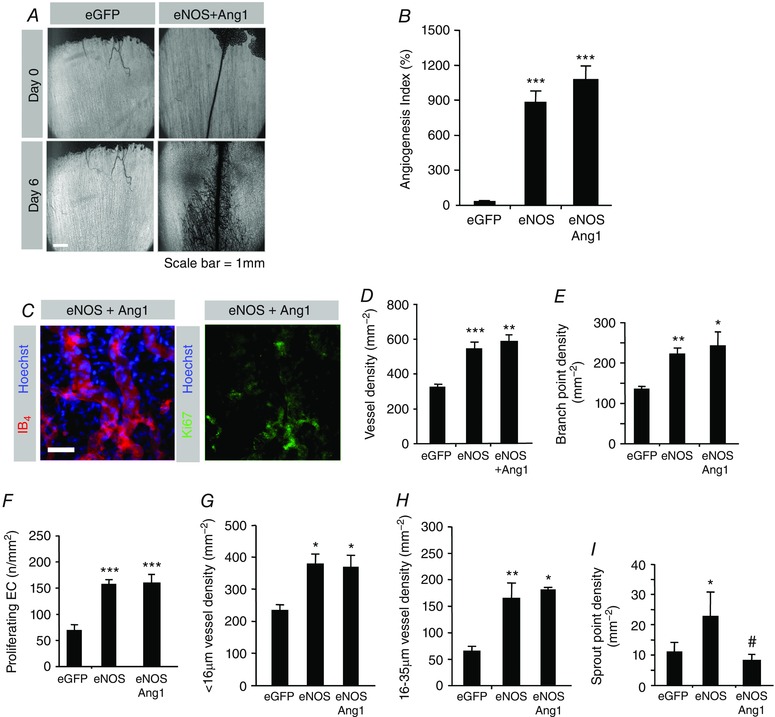
NO–Tie‐mediated angiogenesis is as efficient as NO‐mediated angiogenesis *A*, images of mesenteries after treatment with Ad.*eGFP*, and Ad.*eNOS*/Ad.*Ang1*. *B*, angiogenesis index. *C*, confocal stack images from mesenteric panels stained with isolectin B4 and Hoechst 33324 or Ki67 and Hoechst 33324. *D*–*I*, analysis of vessel density (*D*), branch point density (*E*), proliferating endothelial cell density (*F*), exchange vessel density (*G*), conduit vessel density (*H*) and sprout point density (*I*). ^*^ *P* < 0.05, ^**^ *P* < 0.01, ^***^ *P* < 0.001 compared with GFP; ^#^ *P* < 0.05 compared with eNOS. Scale bar: 40 μm.

As angiopoietin was clearly contributing to the NO‐mediated angiogenesis, we set out to determine whether increasing Ang1 expression could further enhance angiogenesis. Addition of Ang1 to eNOS did not further increase FVA (Fig. 6 *A*) or the angiogenesis index (Fig. 6 *B*) when compared to Ad.*eNOS* alone. It also produced no change in proliferating endothelial cell density (Fig. 6 *C–F*), or overall vessel density (Fig. 6 *D*) and branch point density (Fig. 6 *E*). Interestingly, there was also no change in the frequency of either exchange vessels (Fig. 6 *G*) or conduit vessels (Fig. 6 *H*), which we usually observe upon Ang1 stimulation (Fig. 2 *G* and Benest *et al*. 2006). There was, however, a significant decrease in sprouting induced by Ang1 co‐over‐expression with eNOS, which indicated an alteration in the phenotype of the vascular plexus in response to a synergy between NO and Tie signalling.

### Concomitant NO–Tie signalling stimulates arteriolargenesis

**Figure 7:**
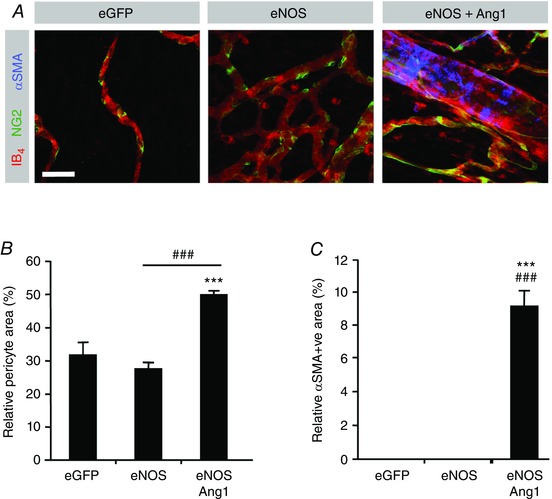
Angiopoietin‐1 is sufficient for NO‐induced arteriolargenesis *A*, sample confocal stack images from rat mesentery 6 days post‐treatment stained with isolectin‐B_4_ and antibodies for NG2 and αSMA. *B*, treatment with Ad.*eNOS*+Ad.*Ang1* significantly increased mural cell coverage. *C*, Ad.*eNOS* was unable to stimulate arteriolargenesis by itself but did so when together with Ad.*Ang1*. Scale bar: 40 μm. ^***^ *P* < 0.001 *vs*. eGFP; ^###^ *P* < 0.001 *vs*. eNOS.

To further evaluate the alteration in phenotype observed upon NO–Tie stimulation, we stained vessels with IB4, NG2 and α‐smooth muscle actin, so that we could determine whether the relative proportions of pericytes and vascular smooth muscle cells (VSMCs) had been altered (Fig. 7 *A*). There was a significant increase in relative pericyte coverage in the Ad.*eNOS*/Ad.*Ang1* co‐transfected mesenteries relative to NO‐mediated angiogenesis alone (Fig. 7 *B*). Smooth muscle actin positive cells with morphology consistent with VSMCs are rarely found in the mesentery of these rats, and none were found in vessels within mesenteries transfected with eGFP or eNOS adenoviruses by themselves, or with Ad.*VEGF*, Ad.*Ang1*, Ad.*sFlt1* or Ad.*sTie2* (not shown). Surprisingly, there was a reproducible and consistent set of vessels staining positive for α‐smooth muscle actin in animals treated with eNOS and Ang1 (Fig. 7 *A–C*). This phenotype was qualitatively consistent with the previously reported arteriolargenic effect achieved upon Ad.*eNOS*/Ad.*Ang1*/Ad.*VEGF* stimulation in this model (around 50% VSMC coverage per vessel area; Benest *et al*. 2008), although the relative VSMC coverage was decreased in this case (around 10%, Fig. 7 *C*).

### Endogenous VEGF signalling is essential for NO–Tie arteriolargenesis

**Figure 8:**
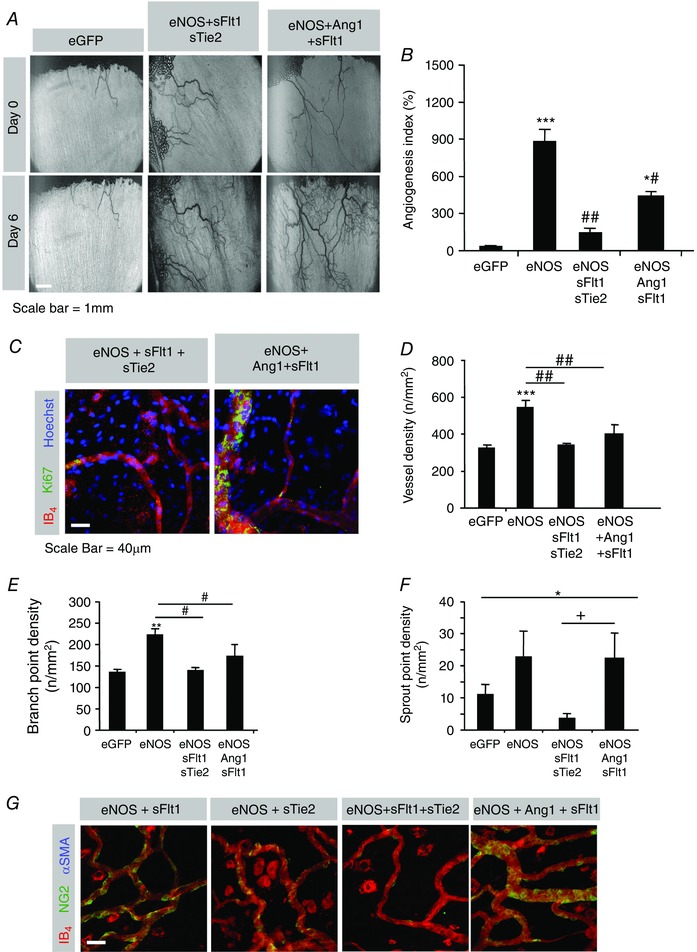
Endogenous VEGF is required for NO‐mediated angiogenesis and NO–Tie‐mediated arteriolargenesis *A*, images of mesenteries after treatment with eNOS with dual VEGF–Ang1 inhibition or eNOS and Ang1 (NO–Tie) with VEGF blockade. *B*, angiogenesis index. *C*, sample confocal stack images from rat mesentery 6 days post‐treatment stained with isolectin‐B_4_, Hoechst 33324 and antibodies for Ki67. *D*–*F*, quantification of vessel density (*D*), branch point density (*E*) and sprout point density (*F*). *G*, images of mesenteries after treatment with eNOS with VEGF inhibition, angiopoietin inhibition, dual VEGF–Ang1 or eNOS and Ang1 (NO–Tie) with VEGF blockade stained with isolectin‐B_4_ and antibodies for NG2 and αSMA. No arteriolargenesis was seen in any of these conditions. ^#^ *P* < 0.05, ^###^ *P* < 0.001 *vs*. eGFP; ^*^ *P* < 0.05, ^**^ *P* < 0.01 *vs*. eNOS; ^+^ *P* < 0.05 *vs*. eNOS–sFlt1–sTie2.

To determine whether the combined effect of NO stimulation and Tie signalling (Ad.*eNOS*/Ad.*Ang1*) was dependent upon VEGF, we analysed the vascular networks formed upon addition of Ad.*sFlt1*. Addition of both Ad.*sFlt1* and Ad.*sTie2* completely blocked NO‐mediated angiogenesis (eNOS+sFlt1+sTie2 compared with eNOS alone, Fig. 8 *A* and *B*), whereas Ad.*sFlt1* reduced by approximately 50% the NO–Tie (eNOS+Ang1)‐mediated angiogenesis (Fig. 8 *B*, compared with Fig. 6 *B*). Staining for Ki67 and isolectin B4 (Fig. 8 *C*) confirmed that the vascular density (Fig. 8 *D*), branching (Fig. 8 *E*) and sprouting (Fig. 8 *F*) induced by NO‐mediated angiogenesis were completely blocked by combined inhibition of both Ang1 and VEGF. However, while both density and sprouting induced by NO–Tie‐mediated angiogenesis were blocked (Fig. 8 *D* and *E*), sprouting was not affected (Fig. 8 *F*). Staining for α‐smooth muscle actin showed that inhibition of either VEGF (eNOS+sFlt1) or Ang1 (eNOS+sTie2) did not induce arteriolargenesis by NO. More importantly, the NO–Tie‐mediated arteriolargenesis (Fig. 7) was not seen when VEGF was inhibited (eNOS+Ang1+sFlt1: Fig. 8 *G*).

## Discussion

The generation of new functional vasculature is a requirement for effective vascular remodelling (Silvestre *et al*. 2013). Previous studies have shown that addition of VEGF can result in more blood vessels whereas addition of Ang1 can result in blood vessels with a more mature structure; neither growth factor alone results in a greater vascular supply with good functionality. The logical option was to combine the delivery of these two growth factors (or a combination of VEGF with another stabilizing agent such as platelet‐derived growth factor B), which resulted in improved cardiac perfusion in a porcine myocardial infarction model (Tao *et al*. 2011) and enhanced perfusion and collateralization (or arteriogenesis) in a rabbit hindlimb ischaemia model (Kupatt *et al*. 2010). However, none of these models had before focused on inducing vasodilatation as a remodelling strategy. We previously showed that inducing vasodilatation can increase the number of vessels, but not the functionality of these vessels. We did find that arteriolargenesis could be generated by combining vasodilatation (either NO or prazosin mediated) with VEGF and Ang1, but not by Ang1 and VEGF alone. Here, we show that arteriolargenesis can be generated by increasing NO production (and hence flow) and administering exogenous Ang1, in the presence of endogenous VEGF signalling. VEGFs generated endogenously during vascular remodelling are necessary for an effective neovasculature. Inhibition of endogenous VEGFs under these circumstances results in a dramatic increase in pericyte recruitment (Fig. 5 *B*) without any increase in vessel diameter or smooth muscle cell recruitment. These results suggest that the method of combining factors that remodels vessels: a) to provide the greatest increase in both exchange and conduit vessels; b) with a proportional increase in vasomotor vessels that can control flow on demand; is to 1) begin by stimulating blood flow, 2) increase Ang1 and 3) allow the vascular system to provide its own VEGFs (which may be optimal at a different kinetics from the other growth factors).

### eNOS‐induced neovascularization is mediated by the VEGF–VEGFR and Ang–Tie axes

A number of studies have highlighted the NO dependency of VEGF‐ (Papapetropoulos *et al*. 1997) and Ang1‐mediated angiogenesis (Babaei *et al*. 2003; Chen *et al*. 2004), and NO has been demonstrated to increase VEGF‐A expression to induce neovascularization. In this model, eNOS over‐expression has been shown to increase levels of endogenous VEGFs by 5‐fold, similar to that induced by Ad‐*VEGF* (Benest *et al*. 2008). Here, the contribution of endogenous VEGFs and angiopoietin signalling to eNOS‐induced angiogenesis was investigated and we confirmed that eNOS‐induced neovascularization is, at least in part, mediated by VEGF–VEGFR. These data are consistent with previous reports demonstrating the VEGF‐A‐dependent action of eNOS in ischaemic tissue (Namba *et al*. 2003; Zhang *et al*. 2003) and upregulation of VEGF‐A in response to Ad.*eNOS* treatment (Benest *et al*. 2008). The difference in sprouting (Fig. 3 *H*) seen between endogenous VEGF expression (the difference between Ad.*eNOS* and Ad.*eNOS*/Ad*.sFlt1*) and exogenous VEGF‐A_165_ expression (Ad.*eNOS vs*. Ad.*eNOS*/Ad.*VEGF‐A_165_*) may therefore be a result of inhibition of the other isoforms, particularly VEGF_121_ and VEGF_189_, which have been shown to regulate sprouting/branching (Ruhrberg *et al*. 2002). Perhaps more interestingly, Ang1 inhibition also inhibited eNOS‐induced neovascularization (AI (Fig. 3 *B*), vessel density (Fig. 3 *D*) and proliferating endothelial cell density (Fig. 4 *B*)). Ad.*eNOS*/Ad.*sTie2*‐treated mesenteries demonstrated substantial haemorrhaging of the newly formed vessels (Fig. 3 *A*), suggesting that inhibition of Ang–Tie signalling appears to disrupt endothelial homeostasis and increase microvessel permeability, potentially leading to a loss of vessel integrity and haemorrhaging. Ang–Tie signalling is known to play an important role in endothelial cell homeostasis (Schubert *et al*. 2011), endothelial cell–cell communication (Fukuhara *et al*. 2008; Saharinen *et al*. 2008) and regulation of microvessel permeability (Thurston *et al*. 2000; Salmon *et al*. 2009). Furthermore, recent work has demonstrated the ability of Ang1 to increase the depth of the endothelial glycocalyx layer in both continuous and fenestrated capillaries, leading to a reduction in water permeability (Salmon *et al*. 2009).

### Blockade of endogenous VEGF–VEGFR or Ang–Tie signalling leads to a phenotypically different angiogenic response

Following treatment with Ad.*eNOS*/Ad.*sFlt1*, EC proliferation was reduced to the level of control (Fig. 4 *B*), subsequently leading to a reduction in exchange vessel density (Fig. 3 *E*), consistent with a role for VEGF‐A in the induction of capillary hyperplasia (Benest *et al*. 2006). Although a reduction in conduit vessel density was induced by Ad.*sFlt1*, this remained 65% higher than control (Fig. 3 *F*), indicating that other factors are involved in the formation of arterioles and higher order vessels. These may include basic fibroblast growth factor (Schierling *et al*. 2009), ephrin‐B2 (Korff *et al*. 2008), delta‐like ligands (Limbourg *et al*. 2007) and Notch 1 (Takeshita *et al*. 2007). Unlike VEGF–VEGFR signalling, blockade of Ang–Tie signalling led to a complete inhibition of proliferation in conduit vessel density and diameter (Fig. 3 *F*), while a minor increase in proliferation was observed in exchange vessels compared with GFP (Fig. 3 *E*). While administration of exogenous Ang1 has been reported to induce luminal expansion (Thurston *et al*. 1999), the neovascular phenotype following inhibition of the Ang–Tie axis has not previously been extensively studied under post‐natal physiological conditions. Inhibition of Ang–Tie signalling using a soluble Tie2 fusion protein has previously been shown to inhibit tumour angiogenesis (Lin *et al*. 1997; Lin *et al*. 1998) and ischaemia‐induced retinal neovascularization (Takagi *et al*. 2003). Although generally considered an antagonist of Ang1–Tie signalling (Scharpfenecker *et al*. 2005), the role of Ang2 in angiogenesis is complex and appears to be context dependent (Brindle *et al*. 2006). However, if we assume that under conditions of non‐pathological blood vessel growth, Ang2 acts as an antagonist of Tie2, then we may consider the phenotype of Ad.*eNOS*/Ad.*sTie2*‐induced vessels to be representative of Ang1 inhibition. These results therefore suggest that endogenous angiopoietin signalling is key to vessel calibre regulation in non‐pathological adult neovascularization.

### Ad.*eNOS*‐induced neovascularization is entirely blocked by combined inhibition of VEGF–VEGFR and Ang–Tie signalling

Although inhibition of either VEGF–VEGFR or Ang–Tie signalling alone was unable to reduce vessel growth to the level of control treated animals (Fig. 3 *B*), blockade of both pathways decreased functional vessel area to control values (Fig. 8 *B*). This is consistent with studies in murine models of retinal ischaemia, where combined intravitreal injection of soluble Flt‐1 and soluble Tie2 fusion proteins led to a significantly greater inhibition of angiogenesis than either treatment alone (Takagi *et al*. 2003). Furthermore, combined blockade of VEGFR2 and Tie2 led to a greater inhibition of tumour angiogenesis than either factor alone (Jendreyko *et al*. 2005). These results therefore support the recent findings that double blockade of Ang and VEGF signalling may be more effective than either alone (Koh *et al*. 2010).

### VEGF‐A inhibits pericyte recruitment but is required for αSMA^+^ cell recruitment

Simultaneous eNOS, VEGF‐A_165_ and Ang1 treatment stimulated an increase in the number of αSMA^+^, VSMC‐type cells in the mesenteric angiogenesis assay (Benest *et al*. 2008). While the role of VEGF‐A in angiogenesis is well defined (Ferrara, 2009), the contribution of VEGF‐A to arteriogenesis and vessel maturation is less clear. Angiogenesis occurs in response to tissue hypoxia, resulting in endothelial cell sprouting and capillary network expansion. However, under conditions of excessive VEGF‐A expression, for example in tumour angiogenesis, a number of studies have demonstrated the induction of tortuous and leaky vessels, with poor mural cell coverage (Thurston *et al*. 1999; Winkler *et al*. 2004; Jain, 2005). In contrast, following induction of ischaemia, inhibition of endogenous VEGF‐A blocks the formation of functional collateral vessels (Jacobi *et al*. 2004).

To clarify the roles of exogenous VEGF and Ang1 in the process of arteriolargenesis we assessed mural cell recruitment. eNOS and Ang1 were capable of increasing both pericytes and VSMCs indicating that exogenous Ang1 but not VEGF‐A is required for arteriolargenesis in non‐ischaemic tissue. Interestingly, inhibition of endogenous VEGFs resulted in a significant increase in relative pericyte coverage following both flow‐mediated and flow–Tie‐mediated angiogenesis (Fig. 5 *B*). This could have been the case if VEGF were to induce vessels that did not have pericyte coverage, but this did not appear to be the case, as the overall pericyte coverage with VEGF alone was not different from control mesenteries (Fig. 2 *H*). These data indicate that both exogenous overexpression of VEGF‐A and induction of expression of endogenous VEGFs inhibit pericyte recruitment, further supporting our findings using Ad.*eNOS*/Ad.*VEGF* and Ad.*eNOS*/Ad.*sFlt1*. Although we demonstrated that exogenous VEGF‐A expression negatively regulates pericyte number, the increase of αSMA^+^ cells was comparable following Ang1–eNOS to that with Ang1–eNOS–VEGF administration (Benest *et al*. 2008). The source of the pericytes and smooth muscle cells is not yet known, but it is either through recruitment or differentiation of existing cells (or recruitment of pericytes, followed by differentiation of pericytes into smooth muscle cells). These results indicate that, in contrast to pericytes, αSMA^+^ cell recruitment or differentiation is not inhibited by overexpression of VEGF‐A in the rat mesentery assay. Surprisingly, following VEGF inhibition with Ad.*sFlt1*, Ang1/eNOS did not increase αSMA^+^ cell numbers (Fig. 8 *G*). This may be due to the fact that different VEGF receptors are necessary for sprouting and for αSMA^+^ cell recruitment or differentiation. It is possible that the required interaction between VEGFR1 and neuropilin 1 that activates phosphoinositide 3‐kinase, thus inducing aortic SMC migration *in vitro* (Banerjee *et al*. 2008), was not promoted. It should also be noted that it is possible that the increase in αSMA^+^ cells could be due to a change in pericyte expression so that pericytes express SMA. However, morphologically the αSMA^+^ cells do not look like pericytes, but like smooth muscle cells, though whether this is recruitment, or differentiation, these experiments cannot determine.

### Limitations

sFlt1 and sTie2 inhibit multiple members of the VEGF and angiopoietin family respectively. sFlt1 inhibits PlGF, VEGF‐B and VEGF‐A and sTie2 will block both Ang1 and Ang2. However, it is likely that VEGF‐A and Ang1 are the predominant growth factors as expression of the other family members has not been described in the mesentery under physiological conditions (Li *et al*. 2001; Cao *et al*. 1997). We also make the assumption that eNOS induces increased blood flow in these animals. We have previously shown this to be the case, and the responses seen with eNOS are mimicked by giving vasodilators such as prazosin, but the caveat should be noted. Finally we are investigating vessel growth in the connective tissue of the mesentery, a relatively sparsely vascularized tissue with low metabolic demand. It is likely that this will not be exactly mimicked in all tissues, but it will be of interest to determine whether similar mechanisms occur in skeletal muscle, brain and other tissues. Finally, we have not distinguished arteriolar capillaries from venular capillaries (both termed 16–35 μm exchange vessels) in the analysis.

### Conclusion

Here we have shown that VEGF inhibition can increase pericyte coverage during angiogenesis, and signalling by endogenously produced VEGFs is required for flow–Tie‐increased vascular smooth muscle cells, with concomitant induction of angiogenesis and arteriolargenesis. Due to mechanistic differences between angiogenesis and arteriogenesis, efficient stimulation of both processes may be problematic as treatments that induce angiogenesis may not induce arteriogenesis and vice versa. It seems likely, given the mechanistic similarities between arteriolargenesis and arteriogenesis that arteriolargenic treatments may induce collateral artery growth, indicating that use of agents such as eNOS, which upregulate endogenous VEGFs, in combination with a vascular maturation factor such as Ang1, may provide a useful treatment strategy for the treatment of ischaemic disease.

## Additional information

### Competing interests

There are no competing interests.

### Author contributions

Experiments were undertaken in the Microvascular Research Laboratories at the University of Bristol. Conception and design of the experiments – O.A.S. and D.O.B. Collection, assembly, analysis and interpretation of the data – O.A.S., J.G.C., P.C.L., E.P., M.J.M. and D.O.B. Funding was provided by grants held by D.O.B., the work was supervised by D.O.B., and animal experiments were undertaken under project license written and managed by D.O.B. The manuscript was drafted and critically revised for intellectual content by O.A.S., M.J.M. and D.O.B. All authors have approved the final version of the manuscript and agree to be accountable for all aspects of the work. All persons designated as authors qualify for authorship, and all those who qualify for authorship are listed.

### Funding

This work was supported by the British Heart Foundation grants (FS/06/038, PG/11/67/29067, BS/06/005) and MRC grant number MR/K013157/1.
